# Vanadate‐Based Fibrous Electrode Materials for High Performance Aqueous Zinc Ion Batteries

**DOI:** 10.1002/advs.202307872

**Published:** 2024-01-04

**Authors:** Qimeng Wang, Jianping Wu, Mingming Wang, Haizhou Yu, Xiaoyan Qiu, Wei Chen

**Affiliations:** ^1^ Key Laboratory of Flexible Electronics (KLOFE) and Institute of Advanced Materials (IAM), School of Flexible Electronics (Future Technologies) Nanjing Tech University Nanjing 211816 P. R. China; ^2^ Department of Applied Chemistry School of Chemistry and Materials Science Hefei National Research Center for Physical Sciences at the Microscale University of Science and Technology of China Hefei Anhui 230026 P. R. China; ^3^ Institute of Advanced Synthesis (IAS) School of Chemistry and Molecular Engineering Nanjing Tech University Nanjing 211816 P. R. China

**Keywords:** cathode, ion doping, large‐scale energy storage, vanadate based material, zinc ion battery

## Abstract

Aqueous zinc‐ion batteries (AZIBs) are considered as attractive energy storage systems with great promise owing to their low cost, environmental friendliness and high safety. Nevertheless, cathode materials with stable structure and rapid diffusion of zinc ions are in great demand for AZIBs. In this work, a new kind of potassium vanadate compound (KV_3_O_8_) is synthesized with fibrous morphology as an excellent cathode material for AZIBs, which shows outstanding electrochemical performance. KV_3_O_8_ exhibits a high discharge capacity of 556.4 mAh g^−1^ at 0.8 A g^−1^, and the capacity retention is 81.3% at 6 A g^−1^ even after a long cycle life of 5000 cycles. The excellent performance of the KV_3_O_8_ cathode is benefited from the structural stability, sufficient active sites, and high conductivity, which is revealed by in situ X‐ray diffraction and various other characterizations. This work offers a new design strategy into fabricating high efficiency cathode materials for AZIBs and beyond.

## Graphical abstract

A kind of potassium vanadate compound (KV_3_O_8_) with fibrous surface is designed, which serves as the cathode for AZIBs. The cathode exhibits a discharge capacity of 556.4 mAh g^−1^ at 0.8 A g^−1^, and a capacity retention of 81.3% at 6 A g^−1^ after 5000 cycles. Such performance benefits from the structural stability, sufficient active sites, and high conductivity of the material.

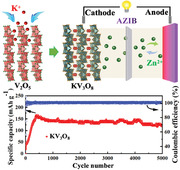

## 1 Introduction

The development of efficient and sustainable energy storage devices is a matter of urgent to satisfy the energy requirements of the growing global population.^[^ ^1^, ^2^ ^]^ Lithium‐ion batteries have played a critical role for decades owing to their high‐energy densities.^[^ ^3^ ^]^ However, lithium‐ion batteries have high cost and safety risks originating from the flammable organic electrolytes, which limit their large‐scale applications.^[^ ^1^, ^4^ ^]^ Scientists are endeavoring in looking for alternative energy storage systems. Aqueous rechargeable batteries are emerging as exciting candidates owing to their improved safety, inexpensiveness, non‐pollution characteristics, and good ionic conductivity compared with the organic electrolytes‐based counterparts.^[^ ^5^ ^]^

So far, a variety of aqueous rechargeable batteries have been studied among which zinc‐ion batteries (AZIBs) have become much attractive as a result of the high theoretical specific capacity (820 mAh g^−1^) and volumetric capacity (5851 mAh cm^−3^),^[^ ^6^, ^7^, ^8^, ^9^, ^10^ ^]^ as well as the low redox potential of the Zn anode.^[^ ^11^ ^]^ In addition, zinc is highly abundant in nature, which could decrease the cost of mass production. However, the cathodes of aqueous zinc‐ion batteries still face challenges such as low capacity and structural collapse, which lead to severe irreversibility of the full battery during the cycling process.^[^ ^12^ ^]^ Therefore, suitable cathode materials are highly demanded to achieve excellent electrochemical performance of AZIBs.^[^ ^13^ ^]^ Most research related to cathode materials for AZIBs mainly focused on manganese‐ or vanadium‐based oxides,^[^ ^14^, ^15^, ^16^, ^17^ ^]^ Prussian blue analogs,^[^ ^18^, ^19^ ^]^ transition metal sulfides, and organic materials.^[^ ^20^, ^21^, ^22^ ^]^

Vanadium oxide stands out among many cathode materials thanks to the high specific capacity and unique layered/tunnel structures for Zn^2+^ intercalation/deintercalation.^[^ ^23^ ^]^ However, the properties of vanadium‐based oxides are degraded due to their rapid dissolution and structural collapse. Several strategies have been recently proposed to address these critical issues, including introduction of metal ions, composite with conductive carbon material or in situ phase transition self‐transformation.^[^ ^24^, ^25^ ^]^ Among them, the introduction of metal ions is more effective from the economic and practical perspective, which could increase the distance between the vanadium oxide layers, thereby significantly improve the cycle performance and rate. For instance, Kundu et al.^[^ ^26^ ^]^ used Zn_0.25_V_2_O_5_·nH_2_O nanosheets as cathodes and achieved excellent rate performance and cycling stability of 1000 cycles. Mai et al.^[^ ^27^ ^]^ found that the pre‐insertion of large ions into layered vanadium oxide facilitated the rapid diffusion of zinc ions and prevented collapse of the layered frameworks during the Zn^2+^ insertion and extraction.

Recently, K^+^ pre‐intercalated V_2_O_5_ was reported as cathodes of AZIBs.^[^ ^28^ ^]^ For instance, Tang et al.^[^ ^29^ ^]^ synthesized K_0.5_V_2_O_5_·0.76H_2_O with exposed surface to enhance the performance of AZIBs. Subsequently, Liu et al.^[^ ^28^ ^]^ assembled a zinc ion battery with highly durable efficiency by introducing K^+^ between nano‐layers of vanadium pentoxide. To date, only few research has been conducted to systematically study the K^+^ ion inserted vanadium oxide as a cathode material, and most K‐intercalated vanadates still exhibited poor electrochemical performance.^[^ ^30^ ^]^ It is highly desirable to develop a more effective K‐intercalated vanadate as a cathode of AZIBs.

Herein, we report a new KV_3_O_8_ cathode material that is synthesized by inserting K^+^ into V_2_O_5_ by a one‐step hydrothermal method, which displays fibrous morphology on the surface. The KV_3_O_8_ cathode displays a high discharge capacity of up to 556.4 mAh g^−1^ when the current density is 0.8 A g^−1^, and the AZIBs achieve an excellent cycling life of 5000 cycles at 6 A g^−1^ with a capacity retention of 81.3%. Moreover, we systematically explore the storage mechanism of the KV_3_O_8_ material by combining in situ X‐ray diffraction (XRD) with transmission electron microscopy (TEM) analysis. It is unveiled that the excellent performance of KV_3_O_8_ is mainly benefited from the synergistic effect of rich active sites, structural stability, and fast zinc ion diffusion.

## 2 Results and Discussion

**Figure 1:**
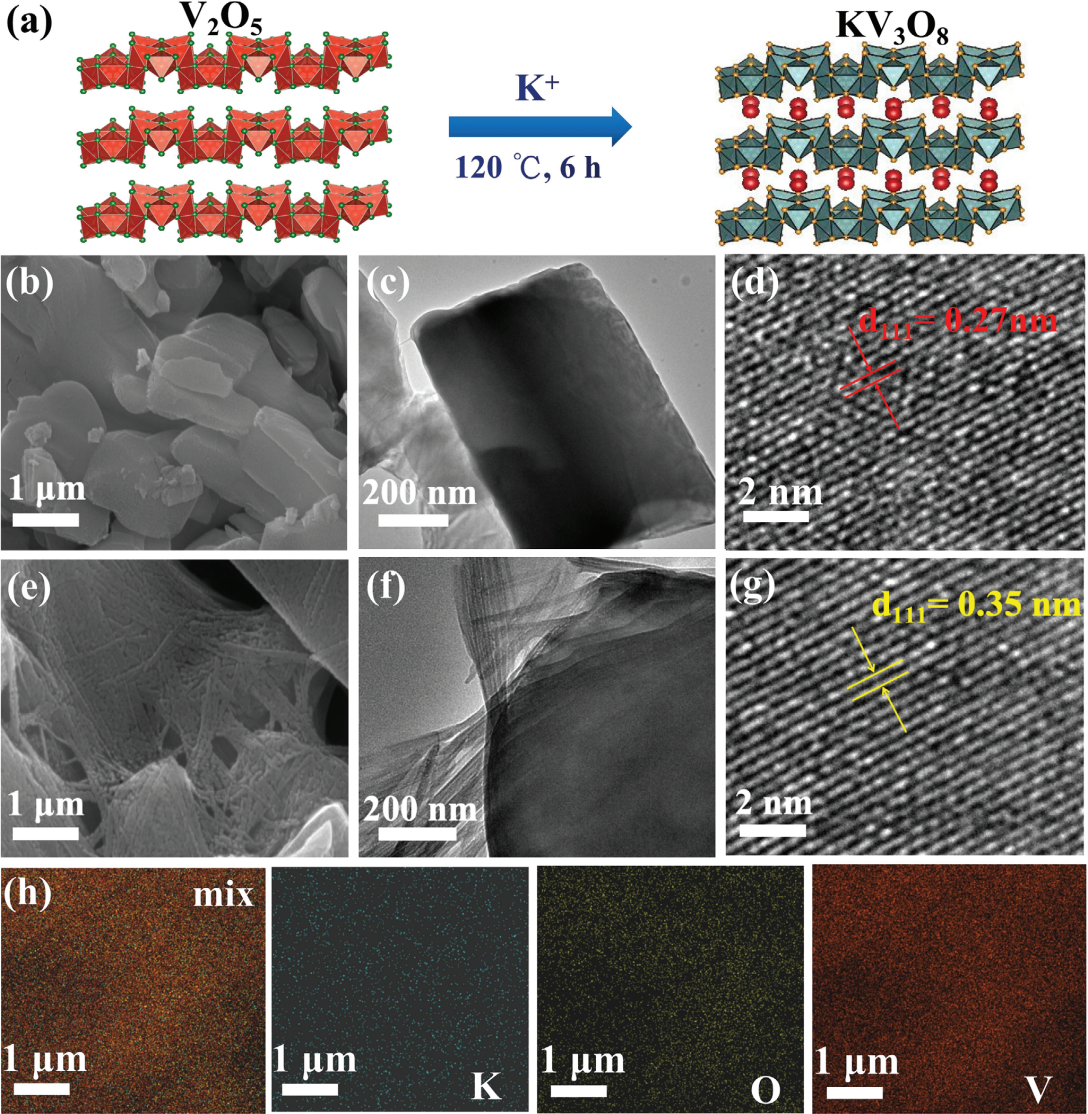
a) Schematic illustration shows the preparation route of K^+^ doping into V_2_O_5_. SEM images of b) V_2_O_5_ and e) KVO. TEM images of c) V_2_O_5_ and f) KVO. HRTEM images of d) V_2_O_5_ and g) KVO. h) EDS elemental mapping images of KVO.

The as‐prepared product was synthesized through a one‐step hydrothermal strategy as described in **Figure** **1**a by inserting potassium ions into the layers of vanadium pentoxide (V_2_O_5_). As evidenced from the scanning electron microscope (SEM) images, the surface morphology changes correspondingly when converted from V_2_O_5_ (Figure 1b) to potassium vanadate compound (KVO) (Figure 1e). It is found that KVO becomes much bumpier compared with V_2_O_5_, and the surface of the KVO appears fibrous.

In this work, the reaction duration between potassium sulfate and vanadium pentoxide plays an important role in determining the morphology of the cathode materials. A series of *ex situ* SEM images indicate that more fibers could be observed when increasing the reaction time. Only a few fibers on the surface were observed after 0.5 h, and no fibers linked between layers (Figure S1, Supporting Information). When it reached 2 h, more fibers could be found. When it is beyond 2 h, the morphology does not change obviously. Therefore, we take 2 h as the optimal condition for the subsequent electrochemical performance characterization, and other conditions for comparison. Figure 1e shows the enlarged image of KVO, which obviously displays a fibrous structure on the surface of KVO. We deduce that the fibers might be formed by stripping from the decomposed material after the reaction between potassium ions and V_2_O_5_. The fibrous features could be evidenced by additional SEM images as shown in Figure S2 (Supporting Information). The successful insertion of K^+^ in V_2_O_5_ could also be verified by the EDS images in Figure 1h.

The structure of KVO was further investigated at different magnifications through SEM and TEM (Figure 1f). The fibers could be clearly observed on the surface of KV_3_O_8_, which can even be formed between layers as support, thereby increasing the stability of the material layers (Figure S2, Supporting Information). The high resolution transmission electron microscope (HRTEM) images indicate that the planar spacing corresponding to the (111) crystal plane is 0.27 and 0.35 nm for V_2_O_5_ (Figure 1d) and KVO (Figure 1g), respectively. This is reasonable since the successful insertion of K^+^ would expand the interplanar spacing. Energy‐dispersive X‐ray element mapping and the energy dispersive spectrometer (EDS) mapping results could further clarify the elemental distribution of the KVO sample. Figure 1h displays the distribution of K, O, and V elements in KVO, demonstrating the successful insertion of K ions in V_2_O_5_. The data from EDS patterns (Figure S6, Supporting Information) indicates a higher potassium content in KVO than that in KVO‐2 or KVO‐3.

**Figure 2:**
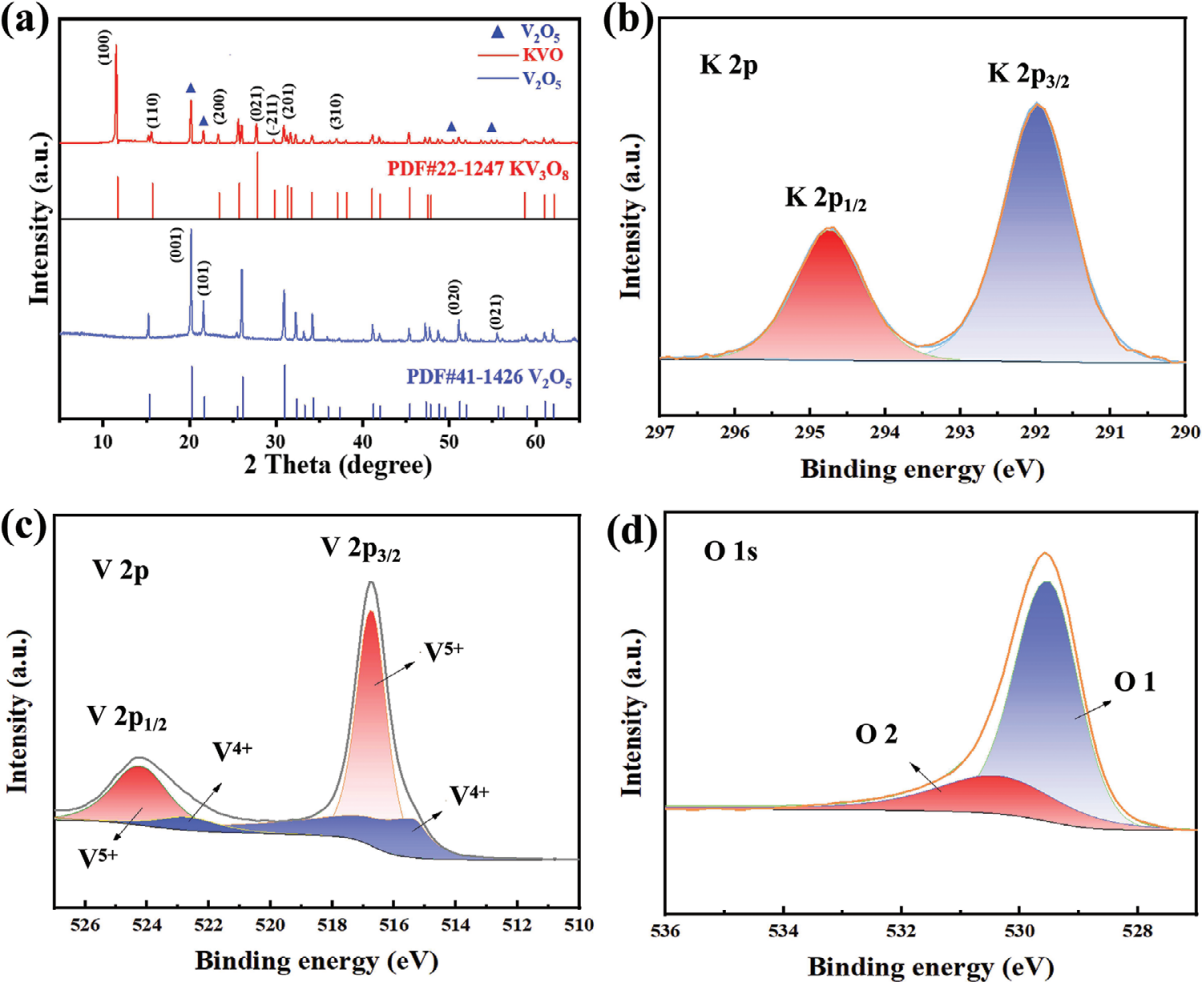
Structural characterizations of KV_3_O_8_: a) XRD patterns, XPS spectra of b) K 2p, c) V 2p, and d) O 1s.

The isothermal curves (Figure S3a,b, Supporting Information) of V_2_O_5_ and KVO from Brunner‐Emmet‐Teller (BET) analysis indicated the narrow parallel channels formed between the layers of the vanadium‐based materials. Figure S3c,d (Supporting Information) illustrates that the specific surface area increased from ca. 9.2 to ca. 10.5 m^2^ g^−1^, which should be attributed to the formation of the fibers. The XRD analysis was implemented to investigate the phase state of the materials. The XRD patterns of KVO (**Figure** **2**a) indicate a mitxure of KV_3_O_8_ and V_2_O_5_. The dominant product is KV_3_O_8_ with a small amount of unreacted V_2_O_5_, and the characteristic peaks of the dominant KV_3_O_8_ agree well with the KV_3_O_8_ phase (JCPDs No. 22‐1247). In this paper, KV_3_O_8_ refers to KVO for clarification.In addition, the XRD spectra of KVO, KVO‐2, and KVO‐3 (Figure S4, Supporting Information) showed an overlap with the characteristic peak of V_2_O_5_, confirming that K^+^ has been successfully inserted into the V_2_O_5_ host material.

To quantitatively analyze the potassium doping amount inductively coupled plasma (ICP) test was performed (Table S1, Supporting Information). The results showed that ca. 6.72 wt.% potassium has been successfully doped in the KV_3_O_8_. The K^+^ insertion is considered to play a role of “pillar” between the layered structure of V_2_O_5_. This can effectively keep the active material from dissolving and enhance the electrochemical performance of the battery.^[^ ^31^ ^]^ ^30^The thermal stability of KV_3_O_8_ was tested by thermal gravimetric analysis (TGA) (Figure S5, Supporting Information). The material could tolerate up to 500 °C. The weight loss from room temperature to 150°C is usually caused by the weakly bound water molecules. Intense water (ca. 4.2 wt.%) was detected between 150 and 200°C. It has been reported that the existence of water in layered oxides might cause more high‐volume transport and ion diffusion, thereby improving structural and electrochemical stability during guest ion intercalation/detachment.^[30,^ ^32^ ^]^

The valence state and chemical composition of K^+^ doped samples have been investigated by X‐ray photoelectron spectroscopy (XPS) analysis. The typical signals for K 2p_1/2_ and K 2p_3/2_ correspond to 294.69 and 291.68 eV (Figure 2b), respectively. This is due to the generation of the K─O chemical bonds, which proves the insertion of K^+^ into the vanadium pentoxide.^[^ ^33^ ^]^ For the V 2p spectrum (Figure 2c), the binding energies of V 2p_3/2_ and V 2p_1/2_ are 516.57 and 524.45 eV, correspondingly, which can be assigned to V^5+^. While the signals at 515.98 and 523.51 eV correspond to V^4+^. Vanadium pentoxide (V^4+^) with mixed valence could enhance the electrochemical activity of cathodes.^[^ ^34^ ^]^ Meanwhile, the signals at 530.1 and 529.38 eV are attributed to O^2‐^ and V─O bonds, correspond to O 2 and O 1 peaks marked in the O 1s spectrum in Figure 2d.^[^ ^35^ ^]^

**Figure 3:**
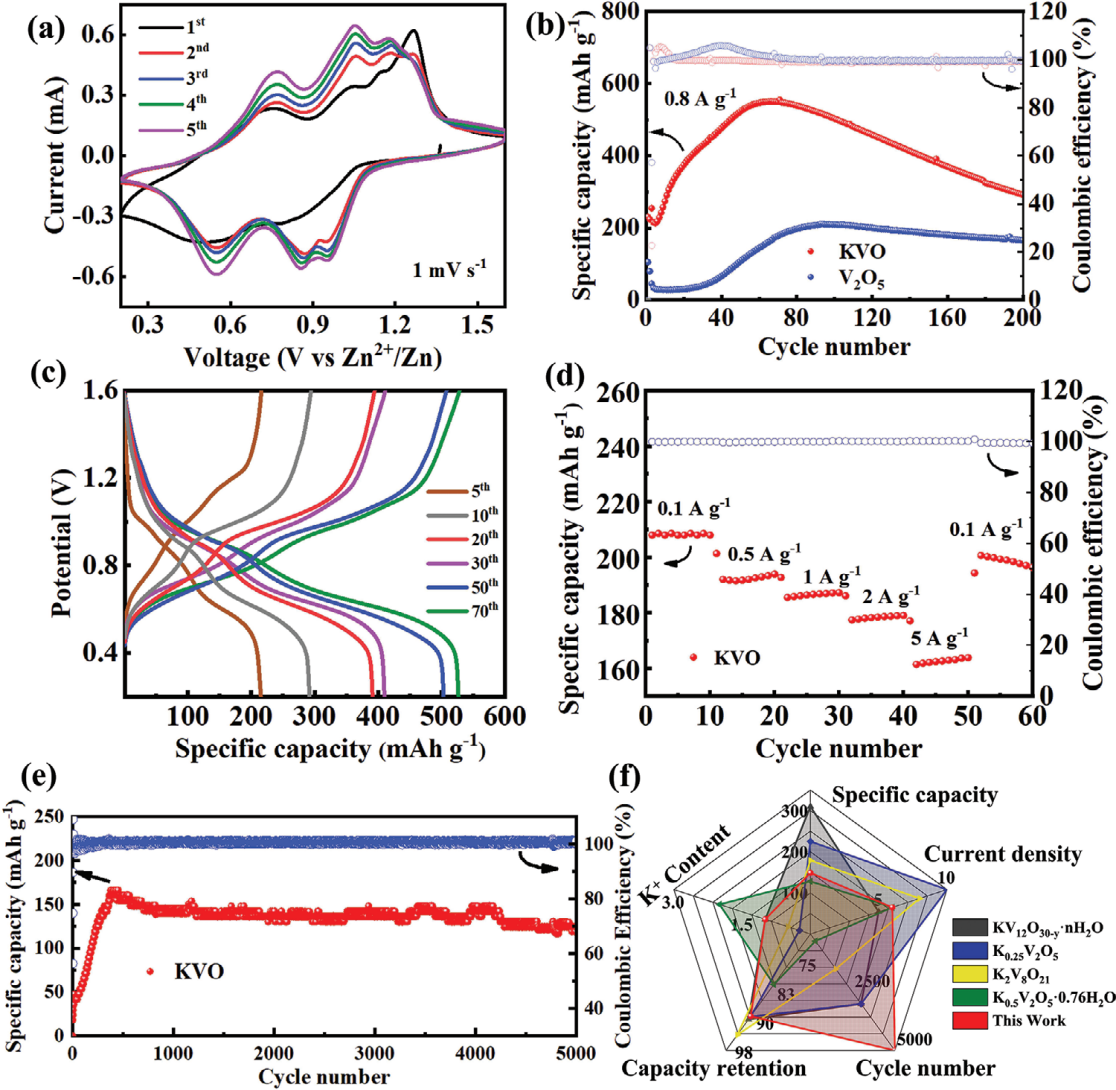
a) CV curves of the initial 5 cycles at 1.0 mV s^−1^ for KV_3_O_8_, b) Cycling life of KV_3_O_8_ when the current density is 0.8 A g^−1^, c) GCD curves of KV_3_O_8_ in different cycles at 0.8 A g^−1^, d) Rate capability of KV_3_O_8_ at diverse current densities, e) Long‐term cycling of KV_3_O_8_ when the current density is 6 A g^−1^, f) Comparison of electrochemical properties of different K^+^ doped materials.

It is anticipated that the exposed layered and fibrous structures of KV_3_O_8_ could provide sufficient active sites as cathode materials. A rechargeable full battery was assembled with KV_3_O_8_ as the cathode material, metallic zinc as the anode, and zinc sulfate as the electrolyte. And a series of electrochemical tests were carried out to evaluate the performance of the AZIBs. **Figure** **3**a exhibits the initial five cycles of the cyclic voltammetry (CV) curve with a scan rate of 1.0 mV s^−1^. The CV curve of the KV_3_O_8_ electrode displays two sets of redox peaks, which are attributed to diverse intercalation/detachment processes of Zn^2+^. The nearly identical curve profiles over different cycles indicate that the electrode material bears a highly reversible cycling performance.

To further evaluate the effects of K^+^ doping on the performance of the battery, Zn/KV_3_O_8_ and Zn/V_2_O_5_ batteries were assembled and tested under the same conditions. Figure 3b shows the cycling performance comparison of Zn/KV_3_O_8_ and Zn/V_2_O_5_ batteries at 0.8 A g^−1^. The discharge capacity of the battery with KV_3_O_8_ cathode is 223 mAh g^−1^ at the beginning. After the 6th cycle the capacity starts to increase until fully activated, and reaches a maximum discharge capacity of 556.4 mAh g^−1^ after the 70th cycle. We hypothesize that the increase of initial capacity might have resulted from the gradual activation of the electrode, which has been well observed from other studies regarding vanadium‐based cathodes.^[^ ^36^ ^]^ However, the Zn/V_2_O_5_ battery has a charge and discharge capacity of only ca. 210 mAh g^−1^ after 95 cycles. This is much lower than the capacity of the Zn/KV_3_O_8_ battery (Figure 3b). It is worth mentioning that the capacity of the Zn/KV_3_O_8_ battery is better than most reported similar cathode materials related to K^+^ intercalation (Table S2, Supporting Information).^[^ ^28^, ^29^, ^37^ ^]^

For comparative analysis, the cycling performances of KVO‐2 and KVO‐3 were tested, which are inferior to that of KVO (Figure S7, Supporting Information) when the current density is 0.8 A g^−1^. The galvanostatic charge/discharge (GCD) curves in Figure S8 (Supporting Information) shows a lower polarization and higher capacity of KVO than KVO‐2 and KVO‐3. And the GCD curve of KV_3_O_8_ shows an obvious charge‐discharge plateau (Figure 3c), which agrees well with the CV curve (Figure 3a). Besides, the GCD curve of pristine V_2_O_5_ at 0.8 A g^−1^ was shown in Figure S9 (Supporting Information). Based on the above results we conclude that the insertion of K^+^ will not change the operating voltage of the material obviously. Therefore, we claim that K^+^ doping could enhance the cycling life and the charging‐discharging capacity of AZIBs.

The rate performance of the batteries with KV_3_O_8_ as the anode material was further tested, which displayed capacities of 210, 190, 185, 180, and 160 mAh g^−1^ at 0.1, 0.5, 1, 2, and 5 A g^−1^, respectively (Figure 3d). When current density was switched to 0.1 A g^−1^ after five different rate tests, the specific capacity returned to 200 mAh g^−1^ with a capacity retention rate of 95.2%. This demonstrated a considerable tolerance of KV_3_O_8_ to Zn^2+^ insertion and the excellent structural stability. The GCD curve of KV_3_O_8_ electrode at various current densities is exhibited in Figure S10 (Supporting Information), which displays evident charge–discharge curves. The electrochemical performance of KV_3_O_8_ was further examined at a current density of 6 A g^−1^ (Figure 3e). The capacity retention rate is as high as 81.3% even after 5000 cycles, indicating the excellent cycling performance of KV_3_O_8_. Such an excellent cycling performance proves that the pre‐insertion of K^+^ is effective and could maintain the integrity of the structure well after long‐term cycling. In contrast, the cathode with pristine V_2_O_5_ could only achieve a retention rate of 18.5% under the same operating conditions (Figure S11, Supporting Information). We also tested KVO‐2 and KVO‐3 after long cycles under the same conditions (Figure S14, Supporting Information). It is obvious that the electrochemical performance of KV_3_O_8_ in this work is better than that of KVO‐2 and KVO‐3. Moreover, we compared the properties of different K^+^ doped materials, and concluded that KV_3_O_8_ showed superior capacity and cycling properties than many other potassium vanadate compounds (Figure 3f). Detailed electrochemical performance data are listed in Table S2 (Supporting Information). Moreover, the cycling performance of the battery with KV_3_O_8_ was examined at other current densities (Figure S15, Supporting Information). Based on these results we envision that the KV_3_O_8_ could serve as a candidate cathode for high‐performance AZIBs.

**Figure 4:**
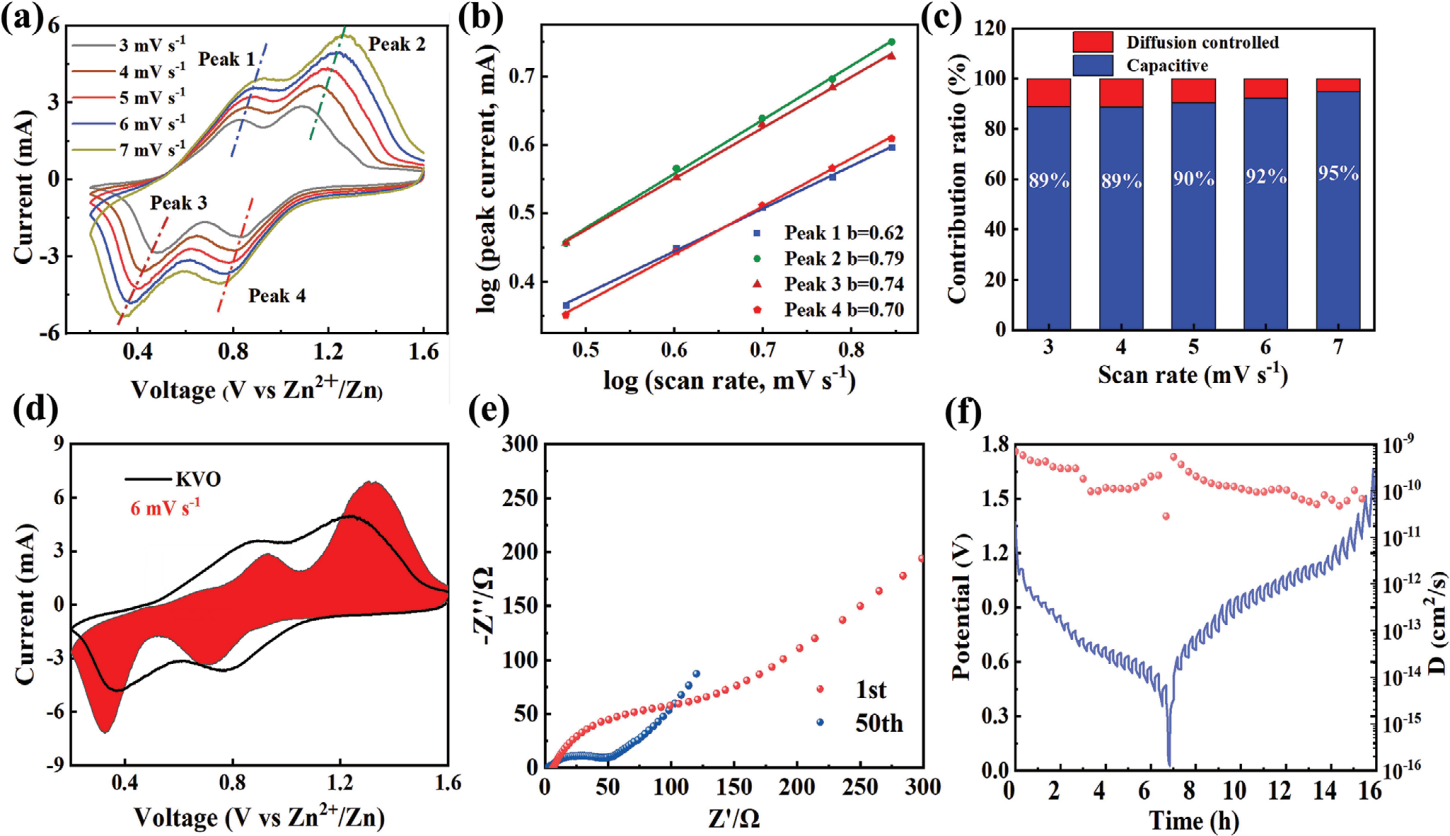
a) CV profiles and b) log (i) versus log (v) of KV_3_O_8_ electrode, c) The capacitance contribution ratio of KV_3_O_8_ at different scan rates, d) A CV curve with capacitance contribution at 6 mV s^−1^, e) Nyquist plots of KV_3_O_8_ electrodes, f) GITT curves of KVO versus Zn^2+^/Zn, and corresponding Zn^2+^ diffusion coefficients during the first charge and discharge process.

In order to study the mechanism of K^+^ insertion for enhancing the electrochemical performance, CV tests were carried out for the KV_3_O_8_ cathode to investigate the electrochemical kinetics. As can be seen from **Figure** **4**a, the CV profiles of KV_3_O_8_ electrode show similar shapes under different scanning rates (3–7 mV s^−1^), including two sets of redox peaks. As the rate increases, the positive peaks move to higher voltages, while the negative peaks shift to lower potentials. The peak shape maintained well at different scan rates, indicating the reversibility and diffusion dynamics of the system. The capacitance‐ and diffusion‐controlled reactions were further quantified to better reveal the reaction kinetics of KV_3_O_8_. In general, the peak current (*i*) and the scan rate (*v*) are described by Equation (1) as following^[^ ^38^ ^]^:

(1) $$i=av^{b}$$

where *a* represents a constant, *b* is derived from the slope of the log (*v*)/log (*i*) curve. When *b* equals to 0.5, ion diffusion is the main process of the reaction. If *b* is between 0.5 and 1, both battery and pseudocapacitive behaviors are exhibited. While *b* is 1.0, it shows a capacitive‐dominated behavior. According to Equation (1), the *b* values at peak 1, 2, 3, and 4 (Figure 4a) are 0.62, 0.79, 0.74, and 0.70 (Figure 4b), respectively.

The capacitive contribution at diverse scan rates could be figured out by Equation (2):

(2) $$i=k_{1}v+k_{2}v^{1/2}$$

where *k_1_v* and *k_2_v* refer to the volumetric and diffusion‐controlled redox reactions, respectively. In addition, when the scan rate elevated from 3 to 7 mV s^−1^, the pseudocapacitive contribution ratio enhanced from 89% to 95% (Figure 4c). For instance, the pseudocapacitive contribution of Zn/KV_3_O_8_ is 92% at 6 mV s^−1^ (red zone in Figure 4d), indicating the fast reaction kinetics and long‐life cycling performance. In Figure 4e, EIS measurements were performed after one and fifty cycles of the KVO electrode. The semicircle in high‐frequency domain and the line in low‐frequency one form a typical Nyquist diagram. The former denotes the charge transfer resistance (*R*_ct_), which is mainly related to the interface between the electrode and electrolyte, while the latter refers to the Warburg impedance (*Z*_w_) of Zn^2+^ diffusion.^[^ ^39^ ^]^ Meanwhile, we tested V_2_O_5_ in the initial state (Figure S12, Supporting Information), and the results indicate that KVO has a lower EIS resistance and charge transfer resistance. The ionic diffusion coefficient (*D*_Zn_) of the cathodes throughout the Zn^2+^ intercalation/deintercalation process was calculated using galvanostatic intermittent titration technique (GITT). Figure 4f shows potential and time curve of the GITT (current pulse). The equation used to calculate *D*_Zn_ is as follows (3):

(3) $$D_{Zn}=\frac{4}{\pi \tau }{\left(\frac{m_{b}V_{m}}{M_{b}S}\right)}^{2}{\left(\frac{\Delta E_{s}}{\Delta E_{t}}\right)}^{2}$$

where *τ* represents the current pulse time (s). *m_b_* is mass (g), and *M_b_* is molecular weight (g/mol). *V_m_* (cm^3^/mol) is the molar volume, and *S* (cm^2^) represents the contact area between electrode and electrolyte. ∆*E_s_* refers to the change in voltage that is caused by current pulse, while ∆*E_t_* is derived from the constant current pulse. In Figure 4f, the *D*_Zn_ of KVO is 10^−10^∼10^−9^ cm^2^ s^−1^, which demonstrates that KVO would promote the rapid migration of Zn^2+^ and lead to the exceptional rate capability. The electrolyte contact angle of the pristine V_2_O_5_ cathodes (Figure S13, Supporting Information) is lower than that of the potassium vanadate compound. This indicates that the formation of KVO after the K^+^ insertion could inhibit the dissolution of vanadium, thus maintain the stability of the cathode, and enhancing the cycling performance.

**Figure 5:**
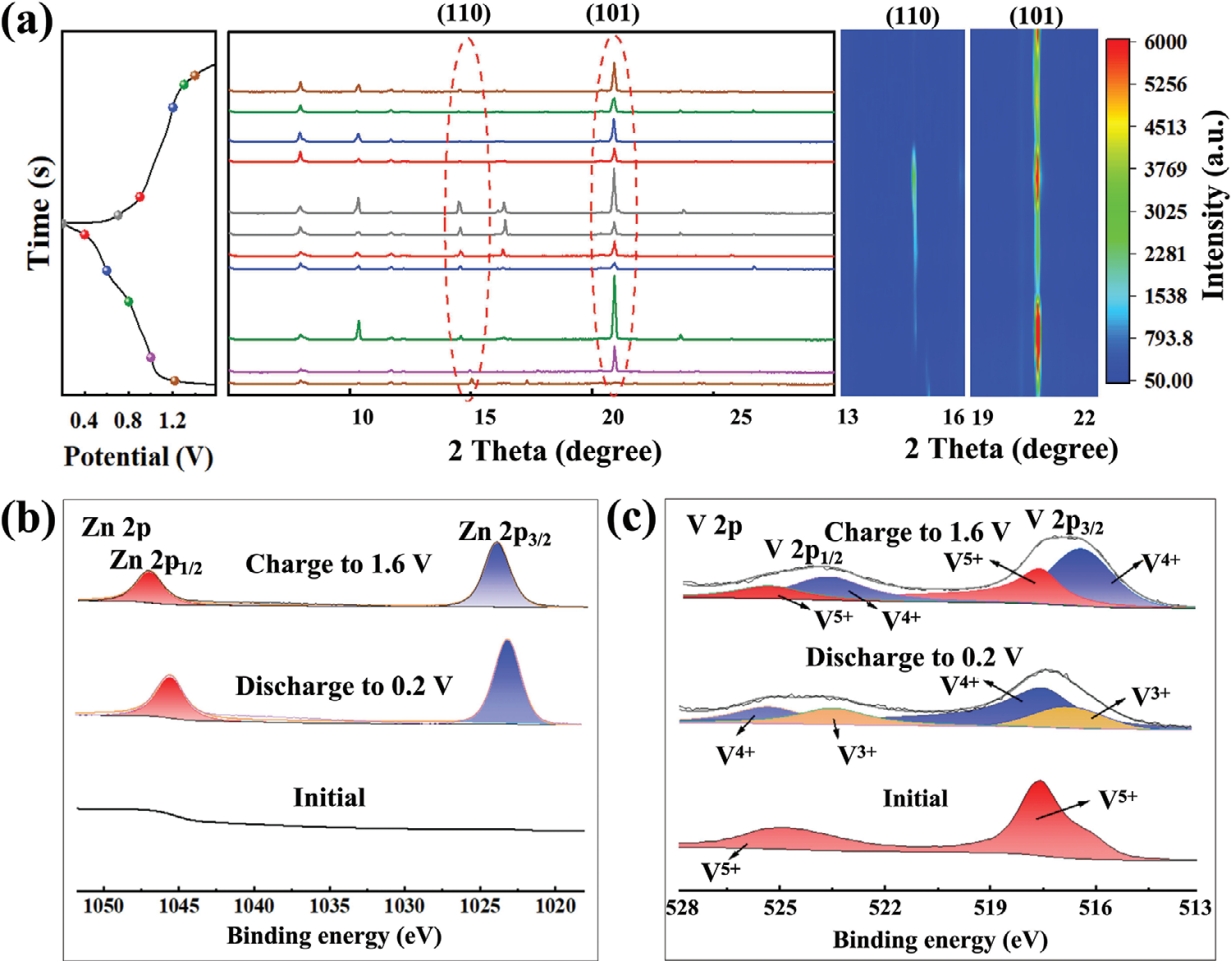
a) The in situ XRD patterns of KV_3_O_8_ when the current density is 0.8 A g^−1^, and the corresponding contour maps of (110) and (101) crystal planes, b) XPS spectrum of Zn 2p. c) XPS spectrum of V 2p.

The electrochemical reaction mechanism during Zn^2+^ storage was further studied by the in situ XRD for KVO electrode (**Figure** **5**a). During different charge‐discharge processes, significant changes happened to the two characteristic peaks at 15.69° and 21.71°. The characteristic peak gradually weakens, and strengthens afterward when discharged to 0.2 V. Such a transition was thought to be resulted from the formation of a new stage (Zn_x_V_2_O_5_·nH_2_O) during the co‐intercalation process of water molecules with Zn^2+^. A similar phenomenon has been reported by the literature.^[^ ^40^ ^]^ The intensity of the diffraction peak gradually converts back into its original state with the embedding of hydrated Zn^2+^ in the subsequent charging process. It should be noted that the diffraction peaks of the (110) and (101) crystal planes slightly shifted to lower angles after discharge and to the initial position in the charged state, corresponding to the extraction and insertion of Zn^2+^. Figure 5a shows a group of reversible profiles that prove the reversibility of the structural change, as well as the local contour maps of (110) and (101) crystal planes. The complete contour map corresponding to Figure 5a is displayed in Figure S16 (Supporting Information).

XPS was then exploited to identify the changes of chemical composition and valence state of the cathode in different discharged/charged states. As seen from Figure 5b, both Zn 2p_3/2_ and Zn 2p_1/2_ exhibit distinct strong signals when fully discharged. On the contrary, the peak intensity reduces when fully charged, which proves the reversible Zn^2+^ insertion/extraction. The signals from the XPS spectrum of V 2p at 517.4 and 524.6 eV in its original state (Figure 5c) refer to V 2p_3/2_ and V 2p_1/__2_ of V^5+^, correspondingly. When discharged to 0.2 V the V^5+^ was reduced to V^4+^ and V^3+^ (Figure 5c), correspondingly. While charged to 1.6 V, the majority of V^4+^ and V^3+^ were reoxidized to V^5+^ and V^4+^, respectively. The reversible intercalation and extraction of zinc ions in the KVO electrode during discharge/charge process was well confirmed.

## 3 Conclusion

We reported in this work a new potassium vanadate compound of KV_3_O_8_, which was successfully synthesized by inserting K^+^ into V_2_O_5_ with a facile hydrothermal method. Such a compound could serve as the cathode for high‐performance zinc ion batteries. The KV_3_O presented a remarkable capacity of 556.4 mAh g^−1^ when the current density is 0.8 A g^−1^, and 81.3% capacity retention was achieved after 5000 cycles at 6 A g^−1^. The charge storage mechanism of KV_3_O_8_ was revealed by combining diverse characterizations, including in situ X‐ray diffraction, XPS and HRTEM, as well as electrochemical analysis. The distinguished electrochemical property of the cathode from KV_3_O_8_ benefited from the exposed layered structure with rich active sites and the high conductivity. We envision that the KV_3_O_8_ cathode material could serve as an excellent candidate for the development of future AZIBs.

## 4 Experimental Section

### Materials Preparation

All chemicals were used directly without further purification. Vanadium pentoxide intercalated with potassium was prepared by a hydrothermal method. Vanadium pentoxide (powder, ≥ 98%, Sigma–Aldrich) and potassium sulfate (ACS reagent ≥ 85%, Sigma–Aldrich) were chosen as the precursors. First, 0.5 mmol potassium sulfate was dissolved in 80 mL deionized water by adding 2 mmol commercial vanadium pentoxide. After vigorously stirring a homogeneous solution was obtained. KVO, KVO‐2, KVO‐3 represented the synthesized potassium vanadate after stirring for 2, 1, and 3 h, respectively, and 2 h was found to be the optimal condition in the present work. Second, the solution was shifted to a 200 mL polytetrafluoroethylene (PTFE) autoclave and reacted at 120°C for 6 h. The materials were then washed and subsequently dried at 60 °C in a vacuum oven. At last, the resulting powder was collected and grounded prior to characterization and testing.

### Structural Characterization

XRD (Rigaku Corporation, Japan) was used to study the crystal structure of the sample. The voltage was 40 kV, and the current was 30 mA (Cu radiation) with a scanning rate of 10° min^−1^. SEM was equipped with a FEI Quanta FEG 250 microscope, and TEM was equipped with a FEI Talos L120C microscope. An EDS analyzer (EMAX Energy EX‐200) was utilized to investigate the elemental distribution. FEI Talos F200X G2 was used to collect HRTEM images. The XPS results were obtained from an AXIS ULTRA DLD. The N_2_ adsorption isotherm was conducted with a Quantachrome Autosorg‐iQ3 analyzer. TGA was executed using the Mettler‐Toledo instrument (Model TG50). The contact angle of the sample was analyzed by Dataphysics OCA20.

### Electrochemical Performance Analysis

A CR2032 coin‐type battery was assembled with zinc foil as anode to study the electrochemical properties of the cathode, which was prepared by blending the active material, polyvinylidene fluoride (PVDF) binder, and acetylene black in N‐methyl‐2‐pyrrolidone (NMP). The resulting slurry was evenly coated on the surface of a titanium foil (0.02 mm in thickness), and dried at 60°C for 12 h. The dried titanium foil was cut into discs (10 mm in diameter). The typical mass loading of the cathode was 1.78 mg cm^−2^. 3 M zinc sulfate was used as the electrolyte. The multi‐channel battery test system (Wuhan Landhe Electronics Co., Ltd., CT3002A) was utilized to carry out GCD tests in a box with a constant temperature of 25 °C. The electrochemical workstation (Shanghai Chenhua Instrument Co., Ltd. CHI 650E) was used to investigate the CV and electrochemical impedance spectroscopy (EIS).

## Conflict of Interest

The authors declare no conflict of interest.

## Supporting information

Supporting Information

## Data Availability

Research data are not shared.
