# Supplementary material for: Vanadate‐Based Fibrous Electrode Materials for High Performance Aqueous Zinc Ion Batteries
Source: Adv Sci (Weinh). 2024 Jan 4;11(11):2307872. doi: 10.1002/advs.202307872 (PMC10953546; doi:10.1002/advs.202307872)
Supplement: Supplementary file 1 — Supporting Information [file ADVS-11-2307872-s001.pdf]

ADVANCED SCIENCE
Open Access

Supporting Information

for *Adv. Sci.*, DOI 10.1002/advs.202307872

Vanadate-Based Fibrous Electrode Materials for High Performance Aqueous Zinc Ion Batteries

Qimeng Wang, Jianping Wu, Mingming Wang, Haizhou Yu, Xiaoyan Qiu and Wei Chen**

Supporting Information

Vanadate-Based Fibrous Electrode Materials for High Performance Aqueous Zinc Ion Batteries

Qimeng Wang, Jianping Wu, Mingming Wang, Haizhou Yu, Xiaoyan Qiu, Wei Chen**

Q. M. Wang, J. P. Wu, Prof. X. Y. Qiu
Key Laboratory of Flexible Electronics (KLOFE) & Institute of Advanced Materials (IAM), Nanjing Tech University, Nanjing, 211816, P. R. China
E-mail: iamxyqiu@njtech.edu.cn

Prof. H. Z. Yu
Institute of Advanced Synthesis (IAS), School of Chemistry and Molecular Engineering, Nanjing Tech University, Nanjing, 211816, P. R. China

M. M. Wang, Prof. W. Chen
Department of Applied Chemistry, School of Chemistry and Materials Science, Hefei National Research Center for Physical Sciences at the Microscale, University of Science and Technology of China, Hefei, Anhui 230026, P. R. China.
E-mail: weichen1@ustc.edu.cn

Supporting Tables

Description of the calculation of data from ICP-OES tests for the content of element K is presented in the following equation:

$$C_x(mg/kg) = \frac{C_0(mg/L) * f * V_0(mL) * 10^{-3}}{m_0(g) * 10^{-3}} = \frac{C_1(mg/L) * V_0(mL) * 10^{-3}}{m_0(g) * 10^{-3}} \quad (1)$$

$$W(\%) = \frac{C_x(mg/kg)}{10^6} * 100\% \quad (2)$$

$$C_1(mg/L) = C_0(mg/L) * f \quad (3)$$

where m_0 is the sample size; V_0 is the volumetric volume; f is dilution factor; C_0 is concentration of the elements of the test solution; C_x is the sample elemental content; W is the elemental content of the sample.

Table S1: ICP results of KV_3O_8.

Sample	m_0 (g)	V_0 (mL)	Element	C_0 (mg/L)	f	C_1 (mg/L)	C_x (mg/kg)	W (%)
KV_3O_8	0.0514	25	K	2.764	50	138.200	67217.90	6.72

Table S2: Comparative electrochemical performance of K^+ doped V-based materials.

	Electrolyte	Specific capacity (mAh g^{-1}; A g^{-1})	Cycling performance	Reaction conditions	Ref.
V_2O_5-nH_2O	$Zn(CF_3SO_3)_2$	>350; 0.3	71%; 900 cycles; 6 A g^{-1}	-	[1]
$K_2V_8O_{21}$	$ZnSO_4$	247; 0.3	83%; 300 cycles; 6 A g^{-1}	180 °C; 48 h	[2]
$K_{0.5}V_2O_5 \cdot 0.76H_2O$	$ZnSO_4$	439; 0.05	96%; 1500 cycles; 8 A g^{-1}	180 °C; 24 h	[3]
$KV_3O_8 \cdot 0.75H_2O$/SWCNT	$Zn(CF_3SO_3)_2$	379; 0.1	91%; 10000 cycles; 5 A g^{-1}	-	[4]
$K_{1.15}V_5O_{13} \cdot 1.3H_2O$	$Zn(OTF)_2$	461; 0.2	99.2%; 4000 cycles; 4 A g^{-1}	-	[5]
$K_{0.25}V_2O_5$	$Zn(CF_3SO_3)_2$	479.8; 0.2	91.3%; 3000 cycles; 10 A g^{-1}	180 °C; 20 h	[6]
$K_{0.23}V_2O_5$	$Zn(CF_3SO_3)_2$	284; 0.1	92.8%; 500 cycles; 2 A g^{-1}	210 °C; 36 h	[7]
$K_{0.25}V_2O_5$	$ZnSO_4$	205; 1	139 mA h g^{-1}; 500 cycles; 2 A g^{-1}	180 °C; 24 h	[8]
$K_2V_8O_{21}$	$LiPF_6$	200.2; 0.05	87.1%; 300 cycles; 5 A g^{-1}	Electrospinning	[9]
$KV_{12}O_{30-y} \cdot nH_2O$	$Zn(CF_3SO_3)_2$	439; 0.05	92%; 3000 cycles; 5 A g^{-1}	120 °C; 6 h	[10]
KV_3O_8	$ZnSO_4$	556.4; 0.8	81.3%; 5000 cycles; 6 A g^{-1}	120 °C; 6 h	This work

Supporting Figures

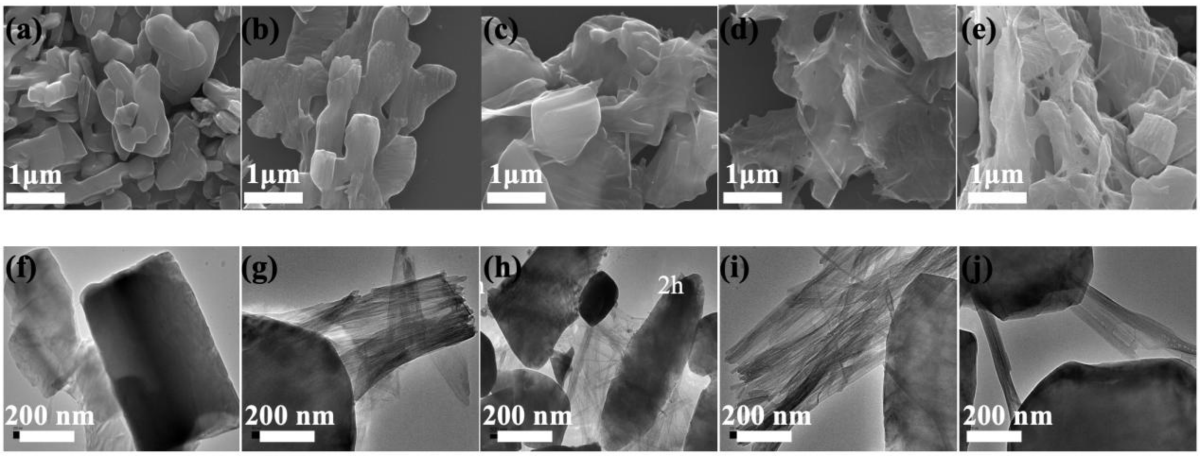

Figure S1. SEM (a-d) and corresponding TEM (f-j) images show the morphological evolution of KVO with the reaction time of potassium sulfate and vanadium pentoxide: (a) and (f) 0.5 h, (b) and (g) 1 h, (c) and (h) 1.5 h, (d) and (i) 2 h, (e) and (j) 2.5 h.

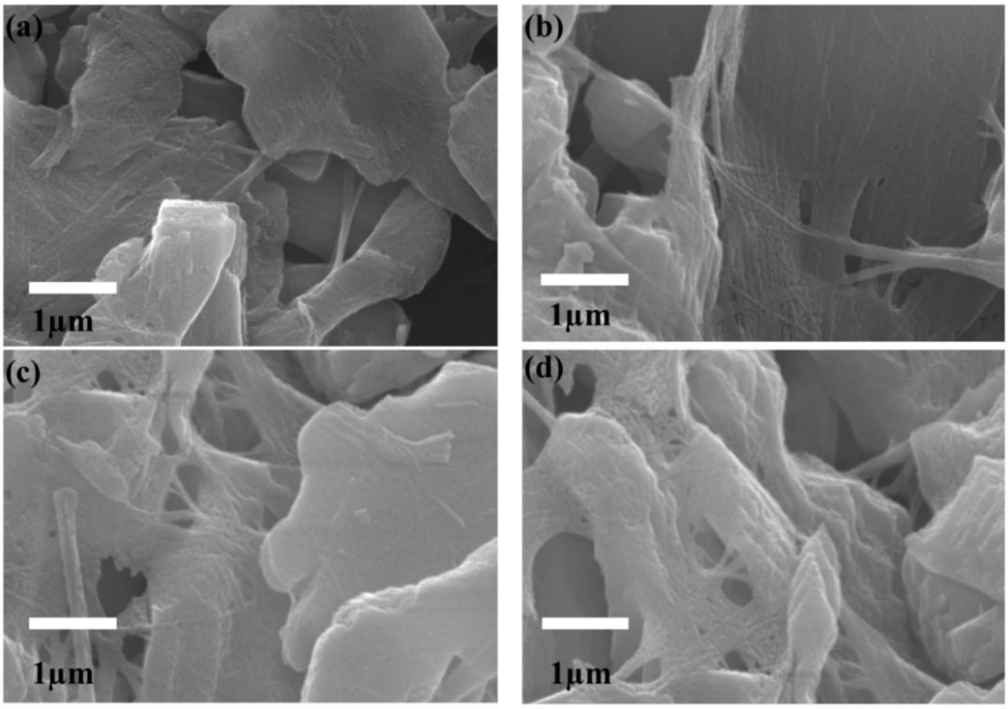

Figure S2. SEM images show the morphology of KV_3O_8.

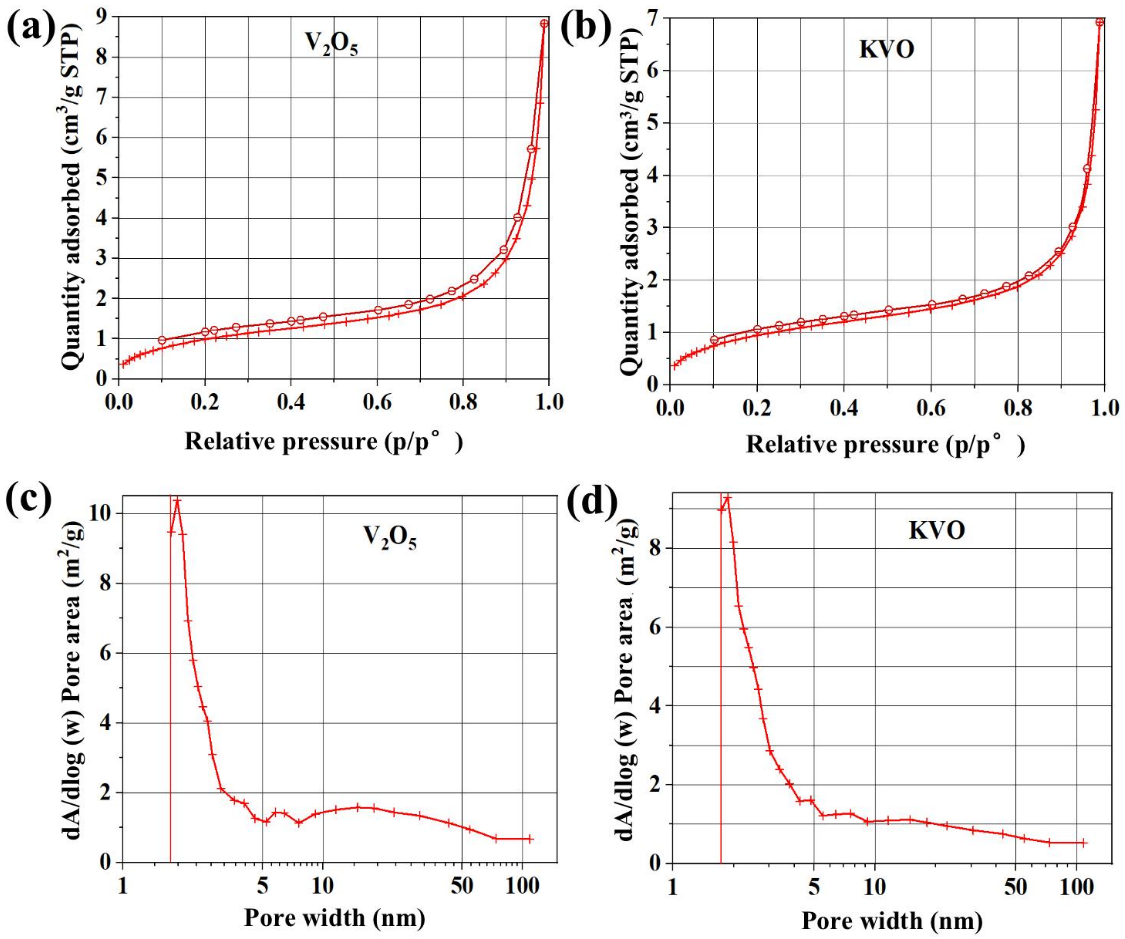

Figure S3. The isothermal curves of V_2O_5 (a) and KVO (b) from BET analysis. The pore width distributions of V_2O_5 (c) and KVO (d).

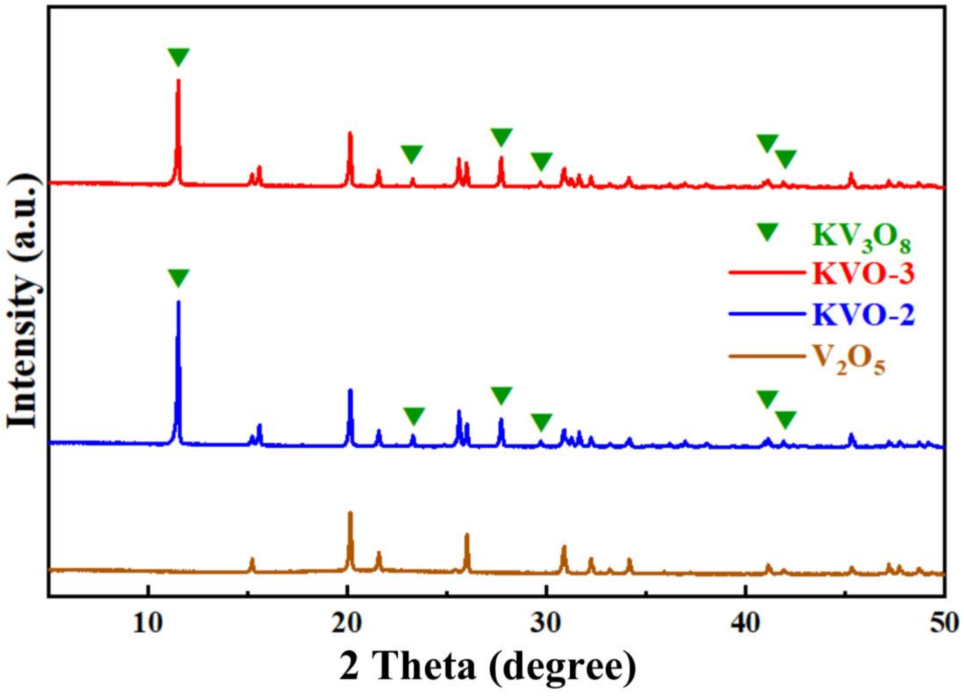

Figure S4. The XRD patterns of KVO-2, KVO-3 and pristine V_2O_5.

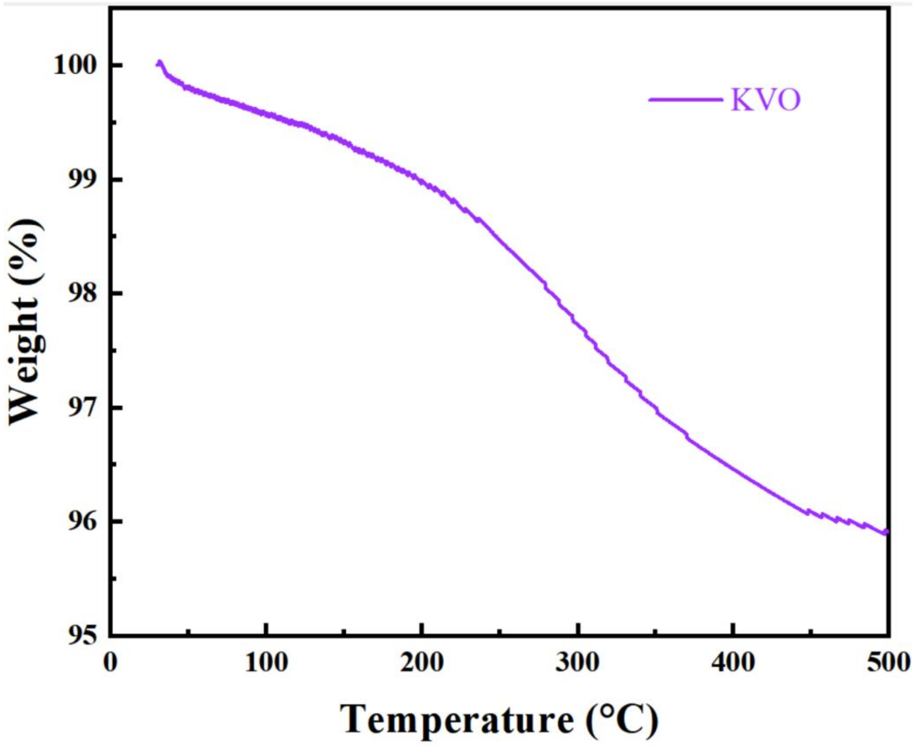

Figure S5. TGA analysis of KVO.

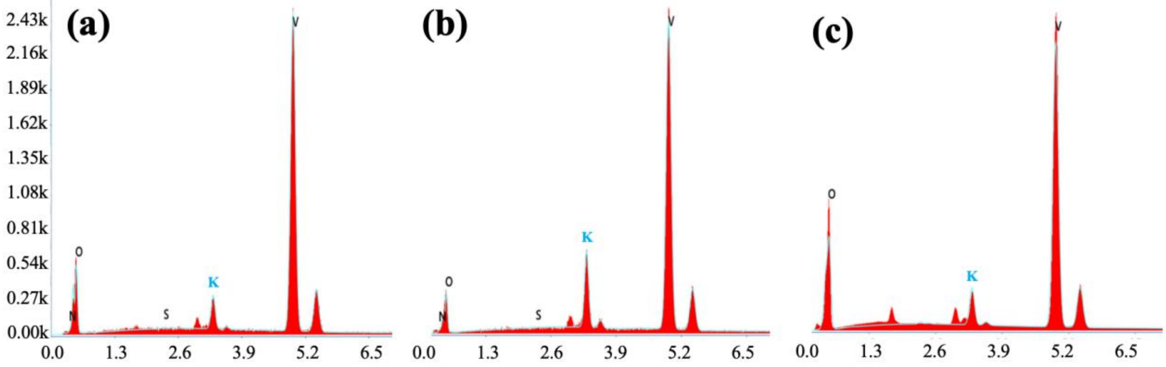

(d)

Element	Ratio (wt%)	Method Detection Limit	Ratio (atom%)	Error%
O	10.6	5.52	26.77	21.71
K	9.54	0.34	9.87	5.48
V	79.86	1.21	63.36	2.63

Figure S6. EDS spectra of KVO-2 (a), KVO (b), and KVO-3 (c). (d) Corresponding atomic ratio of KVO.

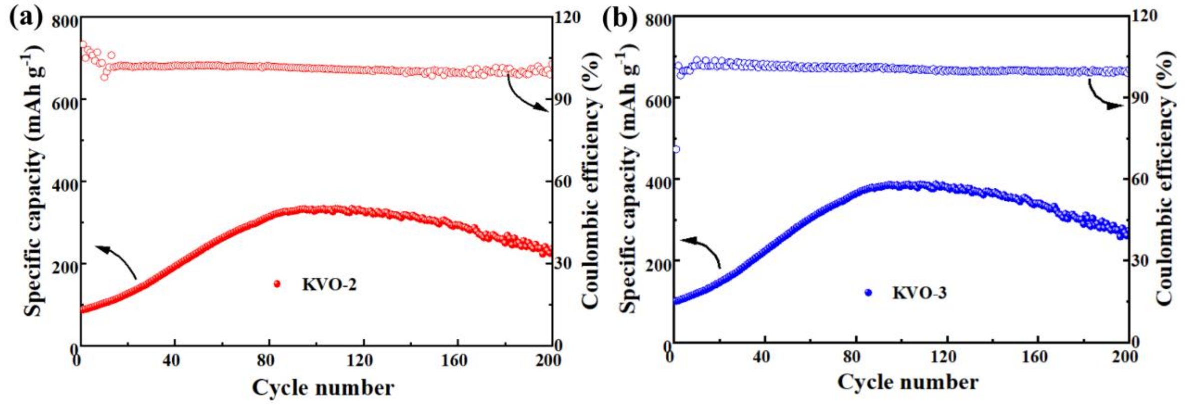

Figure S7. Specific capacity and cycling performance of KVO-2 and KVO-3 at a current density of 0.8 A g^{-1}.

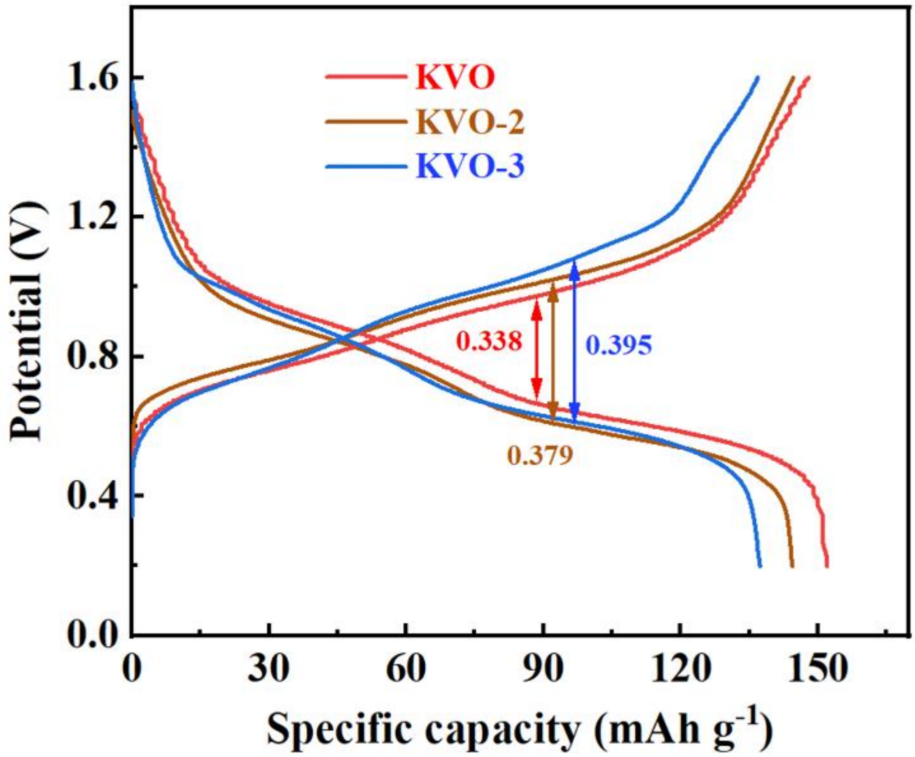

Figure S8. GCD curves of KVO, KVO-2, and KVO-3.

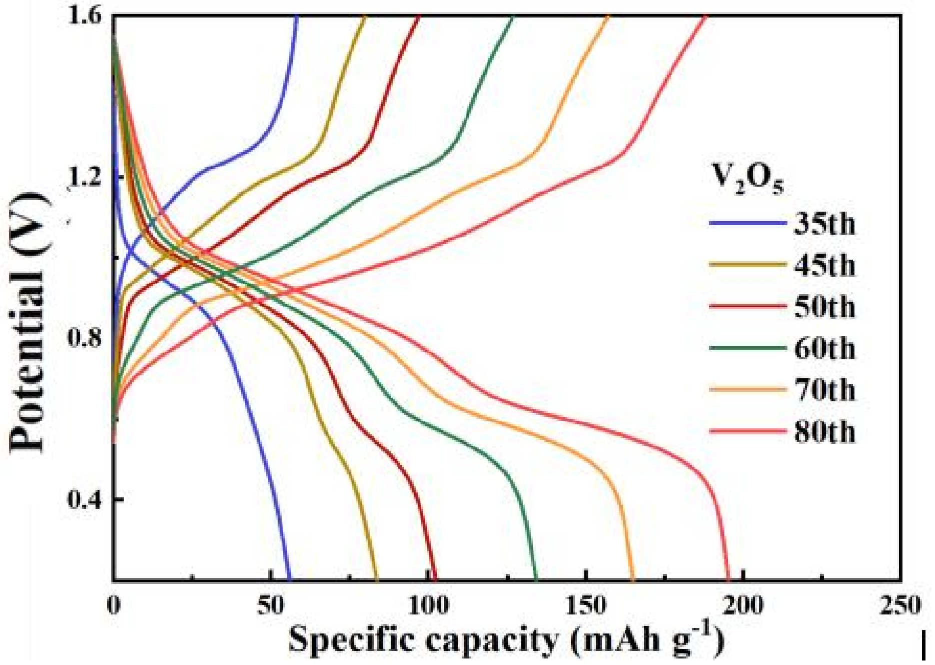

Figure S9. GCD curves of V_2O_5 in different cycles.

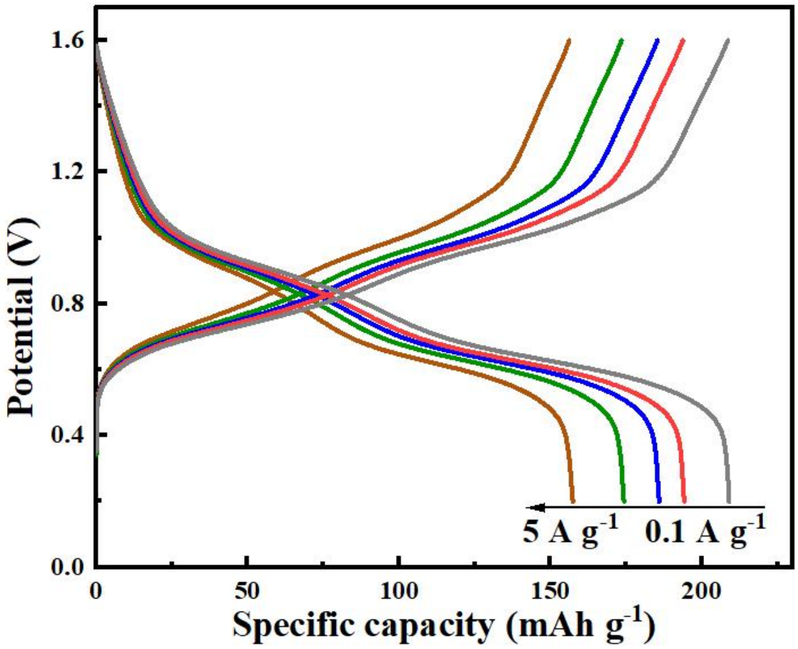

Figure S10. Specific capacity of KV_3O_8 at various current densities.

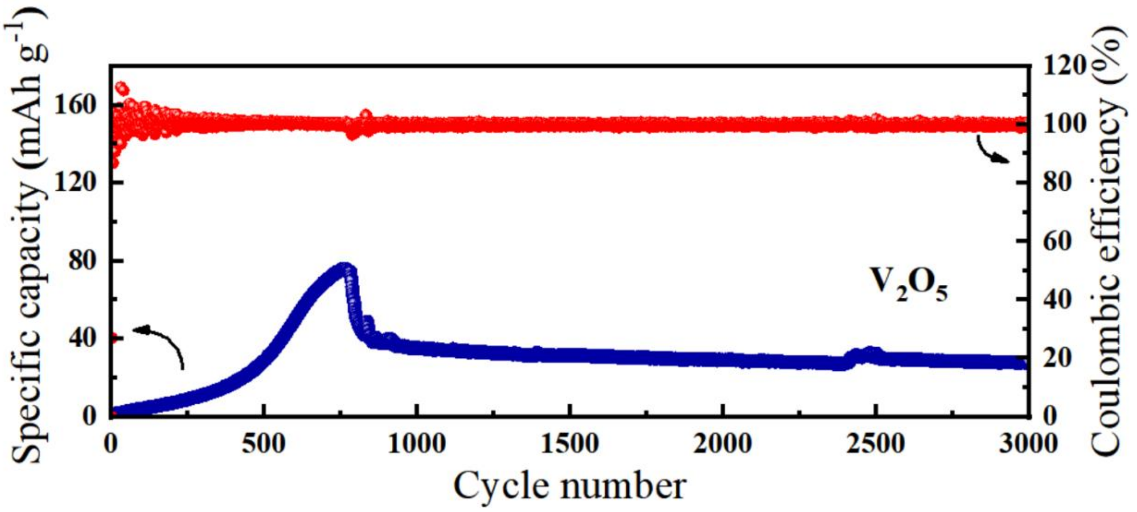

Figure S11. Cycling performance of the pristine V_2O_5.

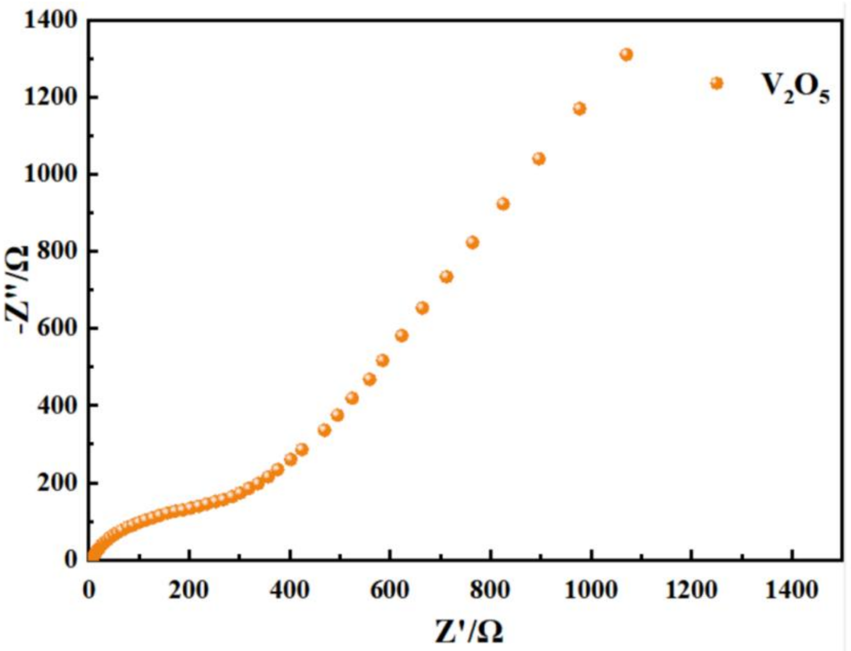

Figure S12. EIS analysis of the pristine V_2O_5.

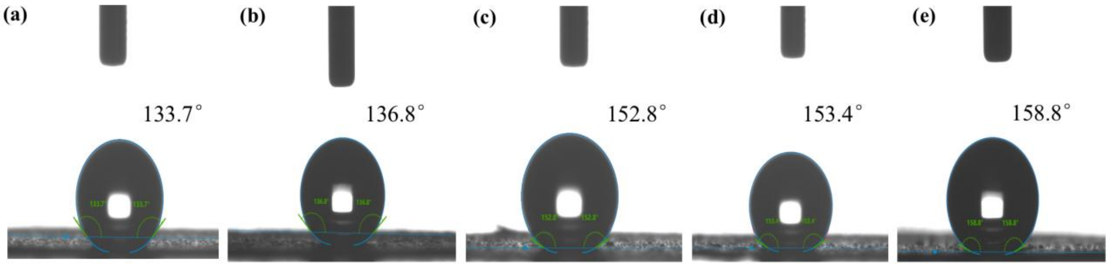

Figure S13. The contact angles of (a) V_2O_5, and KVO synthesized for (b) 0.5 h, (c) 1 h, (d) 1.5 h and (e) 2 h.

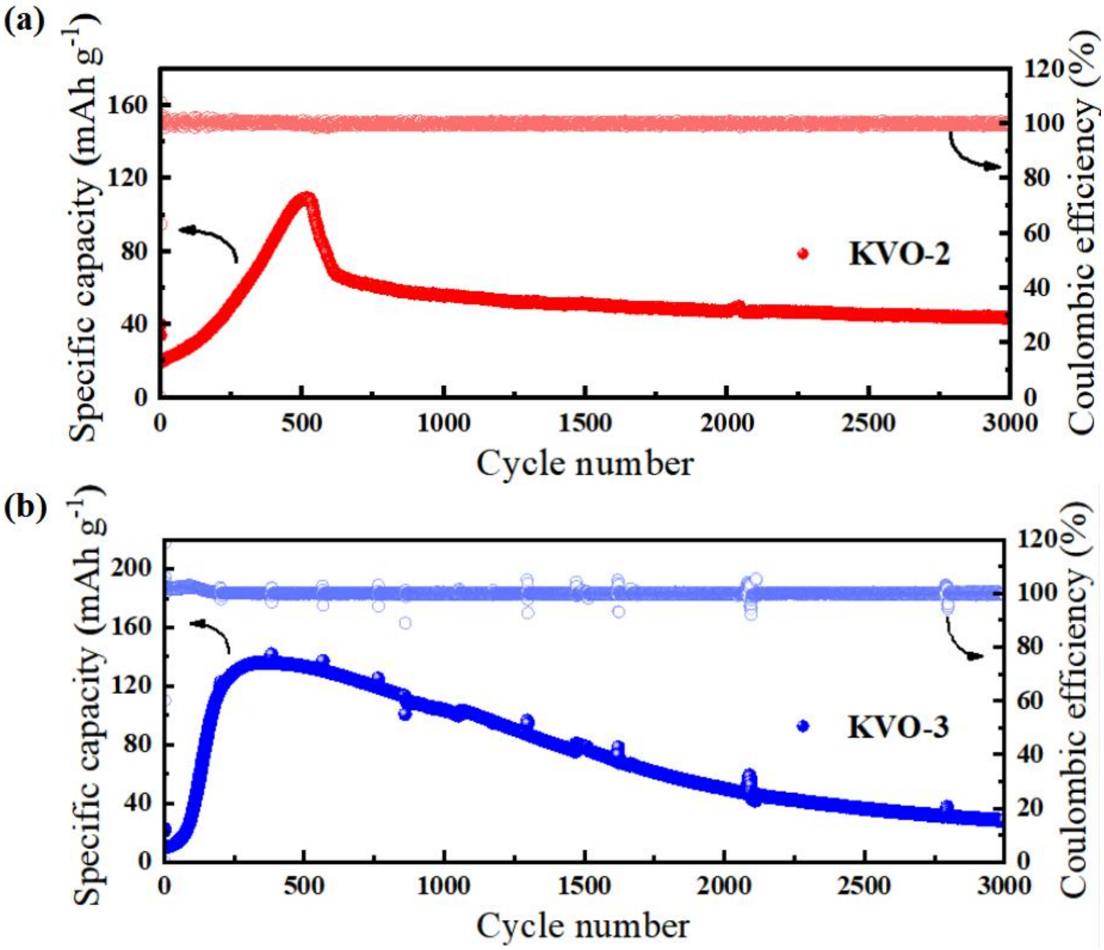

Figure S14. Specific capacity and cycling performance of KVO-2 (a) and KVO-3 (b) at a current density of 6 A g^{-1}.

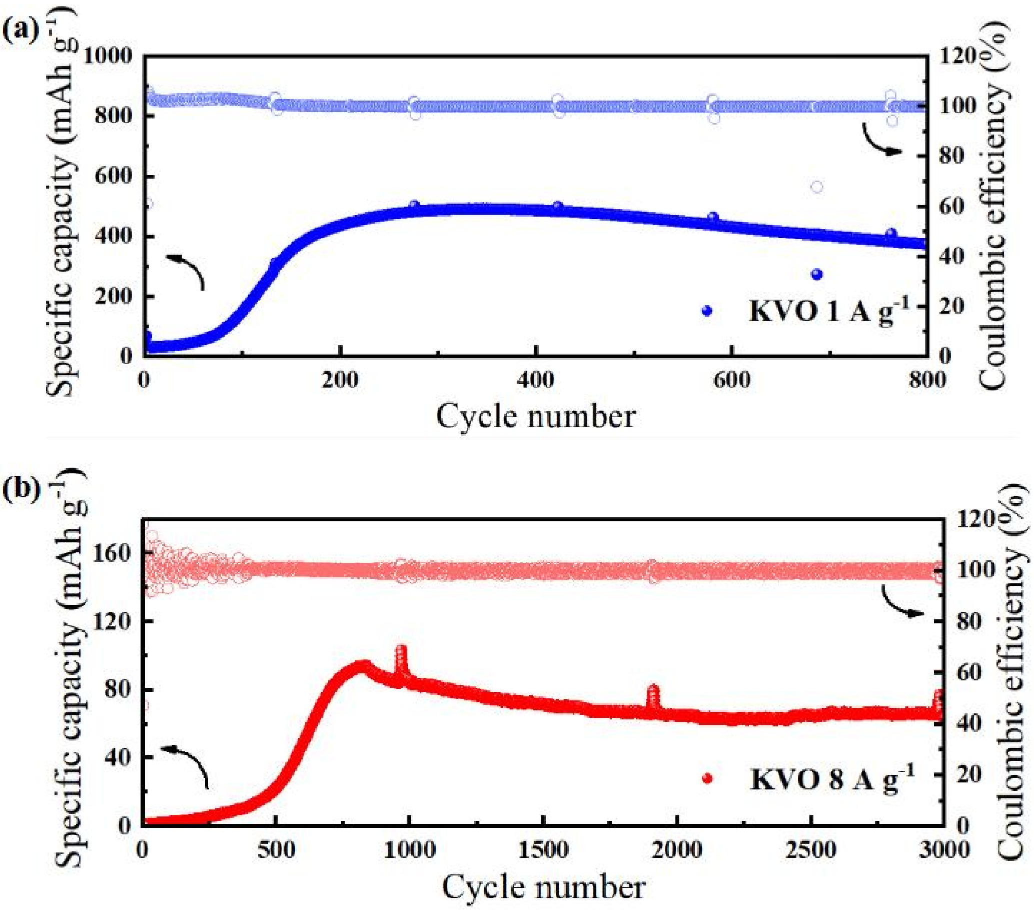

Figure S15. Cyclic curves of KVO at current densities of 1 A g^{-1} (a) and 8 A g^{-1} (b).

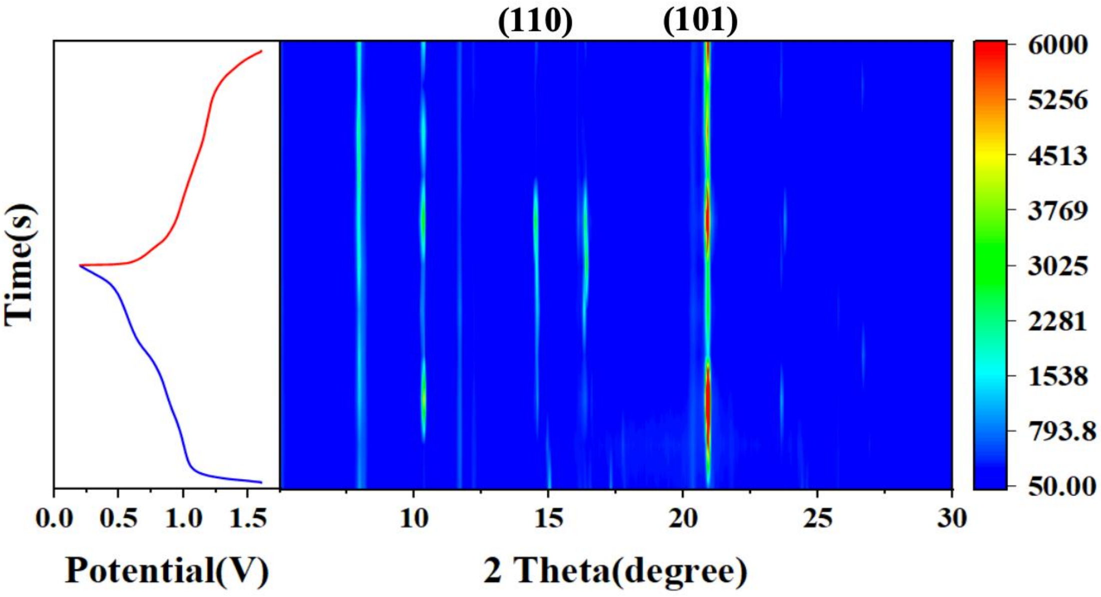

Figure S16. Complete contour map corresponding to the *in situ* XRD spectra.

Supporting References

[1] M. Yan, P. He, L. Mai, J, *Adv. Mater.* **2018**, *30*, 1703725.

[2] B. Tang, G. Fang, J. Zhou, L. Wang, Y. Lei, C. Wang, T. Lin, Y. Tang, S. Liang, *Nano Energy* **2018**, *51*, 579-587.

[3] S. Islam, M.H. Alfaruqi, D.Y. Putro, V. Soundharrajan, B. Sambandam, J. Jo, S. Park, S.Lee, V. Mathew, J. Kim, *J. Mater. Chem. A* **2019**, *7*,20335-20347.

[4] F. Wan, S. Huang, H. Cao, Z. Niu, *ACS Nano* **2020**, *14*, 6752-6760.

[5] N. Qiu, Z. Yang, R. Xue, Y. Wang, Y. Zhu, W. Liu, *Nano Lett.* **2021**, *21*, 2738-2744.

[6] Y. Liu, Y. Liu, X. Wu, Y.R. Cho, *J. Colloid Interface Sci.* **2022**, *628*, 33-40.

[7] W. Zhang, C. Tang, B. Lan, L. Chen, W. Tang, C. Zuo, S. Dong, Q. An, P. Luo, *J. Alloy. Compd.* **2020**, *819*, 152971.

[8] S. Li, M. Chen, G. Fang, L. Shan, X. Cao, J. Huang, S. Liang, J. Zhou, *J. Alloy. Compd.* **2019**, *801*, 82-89.

[9] P. Hao, T. Zhu, Q. Su, J. Lin, R. Cui, X. Cao, Y. Wang, A. Pan, *Front. Chem.* **2018**, *6*,195.

[10] M. Tian, C. Liu, J. Zheng, X. Jia, E.P. Jahrman, G.T. Seidler, D. Long, M. Atif, M. Alsalhi, G. Cao, *Energy Storage Mater.* **2020**, *29*, 9-16.
